# The Pathophysiology of Gastrointestinal and Hepatic Manifestations of COVID-19

**DOI:** 10.7759/cureus.11698

**Published:** 2020-11-25

**Authors:** Muhammad N Yousaf, Haider A Naqvi, Fizah Chaudhary, Kenan Raddawi, Christopher J Haas

**Affiliations:** 1 Medicine, MedStar Union Memorial Hospital, Baltimore, USA; 2 Medicine, MedStar Franklin Square Medical Center, Baltimore, USA; 3 Medicine, MedStar Good Samaritan Hospital, Baltimore, USA; 4 Medicine, MedStar Harbor Hospital, Baltimore, USA; 5 Medicine, MedStar Good Samaritan, Baltimore, USA; 6 Medicine, MedStar Georgetown University School of Medicine, Washington, USA

**Keywords:** coronavirus disease 2019 (covid-19), sars-cov-2 (severe acute respiratory syndrome coronavirus -2), ace2 receptor, gastrointestinal and hepatic manefestations, elevated liver enzyme

## Abstract

Coronavirus disease 2019 (COVID-19), also known as severe acute respiratory syndrome coronavirus 2 (SARS-CoV-2), is the sixth international public health emergency. While COVID-19 classically manifests as a respiratory illness, SARS-CoV-2 may infect multiple organ systems and cause a wide array of presentations. The gastrointestinal tract has become increasingly recognized as a site of SARS-CoV-2 infection with reports of diarrhea, nausea, and liver failure, with or without concomitant respiratory involvement. In this case series and literature review, we report three cases of SARS-CoV-2 infected patients that presented with predominantly gastrointestinal symptoms or laboratory abnormalities such as diarrhea, anorexia, and transaminitis. The receptor for SARS-CoV-2, angiotensin-converting enzyme 2 (ACE2), as well as the necessary protease to facilitate viral entry, transmembrane protease serine-2 (TMPRSS2), and to a lesser extent, cathepsins, have been demonstrated to be present throughout the gastrointestinal tract, thus facilitating viral entry and pathogenesis. Furthermore, multiple reports have demonstrated evidence of viral shedding outside the nasopharynx, including the stool, for prolonged time periods even in the absence of detection of viral RNA in the nasopharynx. As such, testing for SARS-CoV-2 in stool samples with reverse transcription polymerase chain reaction (RT-PCR) assays for detection of viral RNA could aid in identifying patients that lack classic respiratory symptoms, present with atypical symptoms, or in those with a high index of suspicion (e.g. elevated inflammatory markers), but test negative on the classic nasopharyngeal swab. Furthermore, this underscores the potential for atypical transmission, with a focus on fecal-oral transmission and the need for strict hand hygiene.

## Introduction

Coronavirus disease 2019 (COVID-19), also known as severe acute respiratory syndrome coronavirus 2 (SARS-CoV-2), was declared a global public health emergency by the World Health Organization (WHO) and is only the sixth such emergency since the inception of the WHO [1-2]. As of the end of October 2020, over 45 million confirmed cases of COVID-19 have been reported with documented global deaths exceeding 1.1 million [1]. The majority of COVID-19 cases occur in the United States over 8.8 million with estimated deaths exceeding 225,000 and a case fatality rate of 2.2% [1]. This likely represents a striking underestimation of the total number of actual cases when considering the presence of asymptomatic or mildly symptomatic individuals that do not seek medical attention, a high false-negative rate of the nasopharyngeal swab (up to 50%), and the ability of SARS-CoV-2 infection to cause atypical, nonrespiratory presentations. Commonly reported symptoms of COVID-19 are low-grade fever, nonproductive cough, shortness of breath, fatigue, generalized myalgias, anosmia, or hypogeusia [2]. While gastrointestinal symptoms were noted to be the part of the clinical presentation in a proportion of these patients, manifesting as abdominal discomfort, nausea, vomiting, or diarrhea, it has become increasingly recognized that gastrointestinal manifestations may be the sole manifestation of COVID-19 [3]. Of the digestive tract symptoms, anorexia and diarrhea are the most common presenting symptoms followed by nausea, vomiting, mild abdominal pain, and elevated liver enzymes [2, 4-5].

Coronaviruses are important human and animal pathogens. SARS-CoV-2 is an enveloped, single-stranded positive-sense RNA virus that belongs to the same class of other well-recognized coronaviruses including severe acute respiratory syndrome coronavirus (SARS-CoV), Middle East respiratory syndrome coronavirus (MERS-CoV), as well as those strains resulting in the common cold (HCoV-229E, HCoV-NL63, HCoV-OC43, HCoV-HKU1) [5]. SARS-CoV and MERS-CoV originated in bats and subsequently infected humans, breaching interspecies barriers through an intermediate host, civets, and dromedary camels, respectively. SARS-CoV uses angiotensin-converting enzyme 2 (ACE2) as its primary receptor and undergoes receptor-mediated endocytosis via a transmembrane protease serine-2 (TMPRSS2)-dependent proteolytic cleavage process, primarily infecting alveolar type II (AT2) cells and bronchial ciliated epithelial cells [6]. MERS-CoV also infects AT2 cells and bronchial epithelial cells by using different receptors such as dipeptidyl peptidase 4 (DDP4) also known as CD26 [7]. Both SARS-CoV and MERS-CoV infection have been shown to cause gastrointestinal symptoms such as diarrhea in animals and humans [8]. Prior studies have detected SARS-CoV and MERS-CoV from intestinal biopsy and stool specimens, with the presumed replication of these viruses within both small and large intestine resulting in intestinal architectural disruption and associated viral shedding [8-10]. The gastrointestinal manifestations in COVID-19 patients are presumed to be secondary to a similar mechanism.

## Case presentation

Case 1

**Figure 1:**
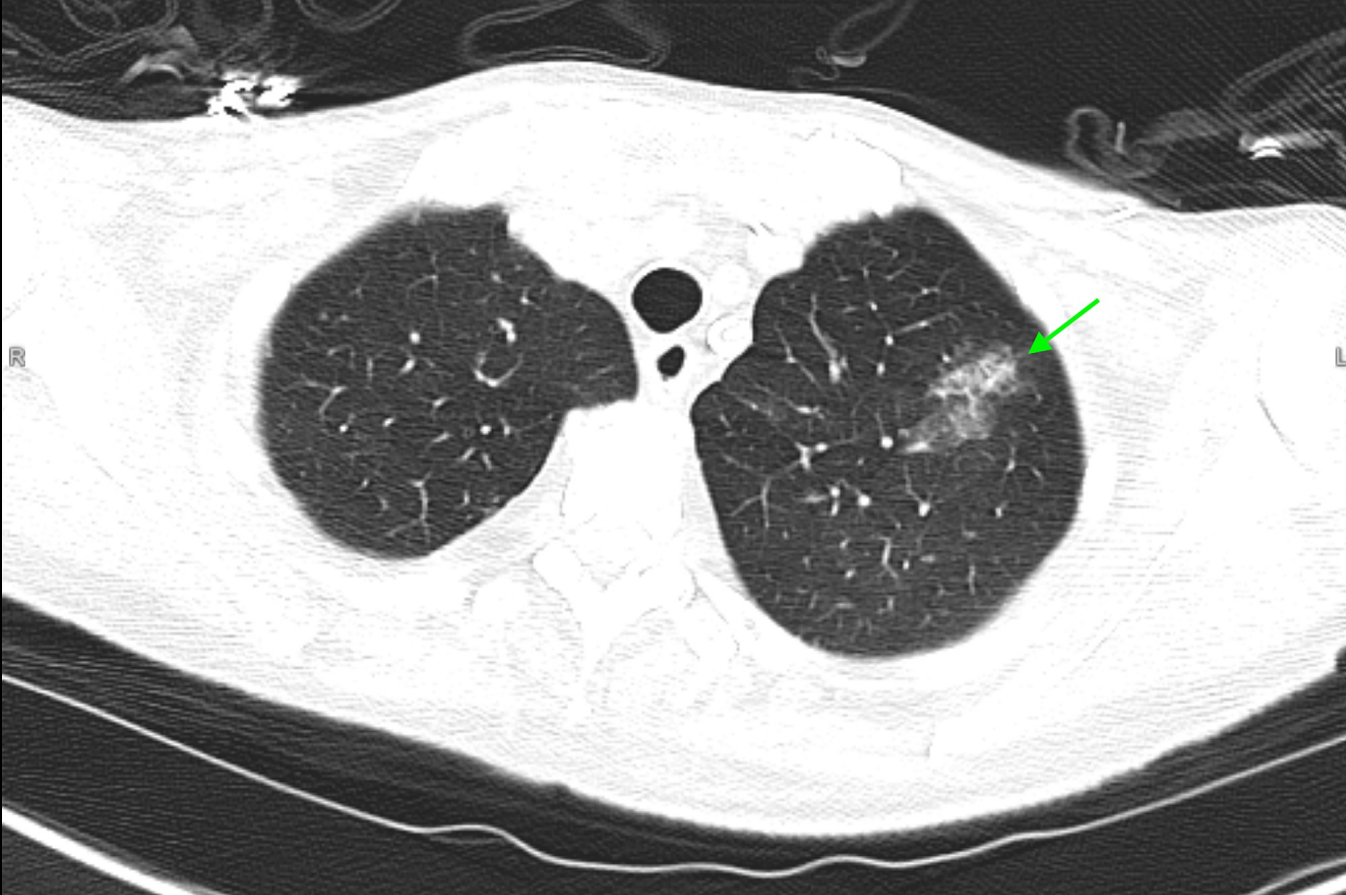
CT chest. CT chest showing a focal ground-glass attenuation in the left upper lobe of lung.

A 92-year-old female with a medical history notable for hypertension, hyperlipidemia, atrial fibrillation on anticoagulation, diabetes mellitus, and chronic kidney disease who presented to the ED with altered mental status, shortness of breath, and abdominal pain of one-day duration. She described diffuse, nonradiating abdominal pain that was crampy in nature, rated 5/10 in intensity, and associated with two episodes of nonbloody and nonbilious emesis. On admission, physical examination was remarkable for elevated blood pressure (188/79 mmHg), but an otherwise preserved heart rate and oxygen saturation. The abdominal examination demonstrated mild discomfort on palpation of the lower abdomen, but no evidence of rebound or guarding. Laboratory workup (Table 1) was remarkable for leukocytosis (19.2 k/uL), an elevated lactate (4.7 mmol/L), elevated C-reactive protein (CRP) (79 mg/L), erythrocyte sedimentation rate (ESR) (60 mm/hour), lactate dehydrogenase (LDH) (281 U/L), creatine kinase (CK) (738 U/L). CT scan of the head and chest radiograph demonstrated no acute abnormalities. CT scan of the chest was subsequently performed and revealed an irregular mass-like area of ground-glass density in the left upper lobe (Figure 1). Given the patient’s overall clinical presentation and elevated inflammatory markers, a nasopharyngeal swab specimen was collected to rule out COVID-19, which ultimately demonstrated SARS-CoV-2 positivity. The patient’s hospital course was complicated by multiple episodes of watery diarrhea for which additional diagnostic workup - stool ova and parasites, stool culture, and *Clostridium difficile* - was ordered and found negative. The patient was treated with supportive care (IV hydration, electrolytes monitoring) and antibiotics which resulted in symptomatic improvement 16 days following presentation and 17 days following onset of symptoms.

**Table 1:** Laboratory diagnostics in patients presenting with gastrointestinal and hepatic manifestations of COVID-19. CRP, C-reactive protein; LDH, lactate dehydrogenase; WBC, white blood cell; ANC, absolute neutrophil count; ALC, absolute lymphocytes count; AST, aspartate aminotransferase; ALT, alanine aminotransferase; NA, not available.

Laboratory test	Reference range	Case 1	Case 2	Case 3
CRP	0-3 mg/dL	79.9	NA	173
Creatine kinase	39-308 U/L	738	160	453
LDH	87-241 U/L	281	NA	757
Ferritin level	28-365 ng/mL	NA	NA	847
WBC count	4-10.8 k/uL	17.1	5.0	18.9
Neutrophil	43%-75%	58.3	72.7	72.1
Lymphocytes	15%-45%	36.7	19.0	10.6
ANC	1.7-8.1 k/uL	10.0	3.6	13.2
ALC	0.6-4.9 k/uL	6.3	0.9	1.9
ANC/ALC ratio		1.6	4.0	6.9
AST	3-38 U/L	23	29	125
ALT	15-41 U/L	17	35	148
Total bilirubin	0.2-1.3 mg/dL	1.0	0.2	0.3
Alkaline phosphate	45-117 U/L	100	82	196

Case 2

A 56-year-old male with a history of obstructive sleep apnea on nightly continuous positive airway pressure (CPAP) presented to the ER with fever and diarrhea for one day and worsening dyspnea of two days duration. He reported persistent day-time dyspnea both at rest and upon ambulation that was associated with an occasional nonproductive cough and sore throat. On the day of the presentation, he developed a low-grade fever and two to three episodes of watery diarrhea. He denied nausea, vomiting, abdominal pain, jaundice, change in his diet or recent travel, however, did report a recent sick contact, a business partner, who had similar symptoms and was found to be SARS-CoV-2 positive. On admission, vitals were unremarkable except for fever to 38.8°C. Clinical examination was normal with the exception of bilateral diminished breath sounds and mild rhonchi. Laboratory workup was unremarkable (Table 1). Chest radiograph showed bilateral patchy infiltrates. He was tested for COVID-19 using a reverse transcription polymerase chain reaction (RT-PCR) assay on a nasopharyngeal swab specimen and was found to be positive for COVID-19. He was treated with supportive care (vitals and electrolytes monitoring, IV hydration) and hydroxychloroquine and azithromycin, despite the lack of convincing clinical evidence. The patient’s symptoms improved four days following presentation and seven days following onset of symptoms. He was discharged home with instructions to continue to self-isolate.

Case 3

**Figure 2:**
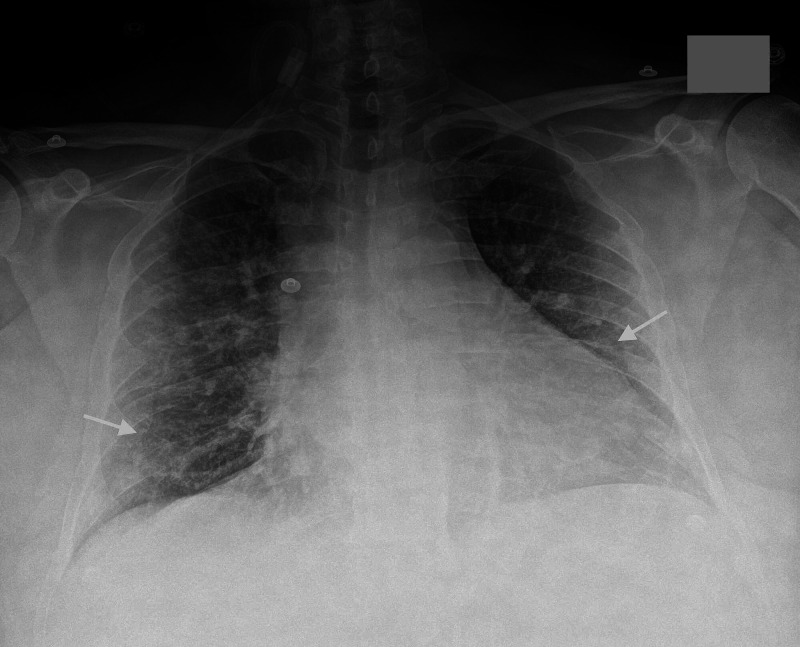
Chest radiography. Chest radiograph showing bilateral diffuse opacities more prominent in the lower lobes (arrows).

A 28-year-old male with a past medical history of insulin-dependent diabetes mellites presented to the ER with fever, dyspnea, and a nonproductive cough of two weeks that had progressed during that time. His symptoms were associated with occasional episodes of light-headedness and reported confusion. He denied sick contacts or illicit substances, tobacco, or alcohol use. On admission, he was febrile to 38.1°C, tachycardic (151/minute), tachypneic (55/minute), and hypoxic with an oxygen saturation of 60% on room air but had preserved pressures. Physical examination was significant for an ill-appearing, diaphoretic man with increased work of breathing. Abdominal examination was unremarkable with no tenderness, rebound, guarding, and noted normoactive bowel sounds. Laboratory workup (Table 1) was remarkable for leukocytosis (18.9 k/uL), an elevated CRP (173 mg/L), ESR (54 mm/hour), ferritin (847 ng/mL) that peaked at 3003 ng/mL on hospital day 10, fibrinogen 655 mg/dL, LDH (757 U/L), CK (453 U/L), and D-dimer (>20 mcg/mL). There was evidence of direct hepatocellular injury with elevated aspartate transaminase (AST) (125 U/L), and alanine transaminase (ALT) (148 U/L), with normal alkaline phosphatase (196 U/L) and total bilirubin (0.3 mg/dL). The viral panel and nasal swab for SARS-CoV-2 were positive. Chest radiograph demonstrated bilateral opacities prominent on lower lobes consistent with COVID-19 (Figure 2). The patient was treated with remdesivir, convulsant plasma, and ventilatory supportive care with subsequent improvement in his symptoms and liver function.

## Discussion

In this case series, we present three patients with laboratory confirmed SARS-CoV-2 infection who presented with gastrointestinal symptomatology or evidence of direct hepatocellular injury on laboratory workup. In the original SARS-CoV epidemic, diarrhea was reported in 10%-20% of patients, whereas the rate was noted to be as high as 30% in those infected with MERS-CoV [8, 11]. Two large meta-analysis have shown an estimated prevalence of gastrointestinal manifestations or liver injury in a fair proportion of cases, 20% and 15%, respectively [5, 12]. In order to infect cells and its downstream effects, these positive-sense RNA-based enveloped coronaviruses must bind to cell surface receptors in order to undergo receptor-mediated endocytosis and subsequent cellular entry. In the case of SARS-CoV-1 and SARS-CoV-2, full-length next-generation molecular sequencing has demonstrated over 80% homology between SARS-CoV-1 and SARS-CoV-2 with nearly identical sequences in the highly conserved spike glycoprotein, S-protein, which plays a critical role in binding to the functional receptor, ACE2 [13]. The S-protein, however, requires priming by another cell-surface protein, TMPRSS2, a protease exploited by multiple pathogenic coronaviruses, as well as influenza [14]. TMPRSS2 cleaves the S-protein into two subunits, S1 and S2, with the S1 carboxy-terminal domain (S1-CTD) functioning as the receptor-binding domain [14]. Intriguingly, even in the absence of TRMPSS2, SARS-CoV-1 and SARS-CoV-2 may still enter into the cells, presumably due to the protease-mediated cleavage by cathepsins [15].

Prior studies demonstrated that both ACE2 and TMPRSS2 are expressed in multiple gastrointestinal cell subtypes including endothelial cells of the liver, as well as the epithelial cells of the esophagus, stomach, duodenum, jejunum, and ileum [14, 16]. Expression was also observed in the epithelial cells comprising the bile canaliculi and ducts (cholangiocytes) but not of hepatocytes [16]. Concurrently, SARS-CoV-1 RNA was detected in the aforementioned tissues but more dramatically, viral particles were observed not only in intracellular vesicles, but also on the luminal surface of enterocytes, suggesting active viral replication, packaging, and release from these cells [8, 16]. As a corollary, SARS-CoV-1 was detected in the stool of the infected patients [8, 10]. The hypothetical fecal-oral transmission seemed quite plausible in the setting of SARS-CoV-1 infection linked to a faulty sewage system [9-10]. Similarly, for SARS-CoV-2, multiple studies demonstrated SARS-CoV-2 isolation from the stool of infected patients with continued viral detection in the stool (up to 33 days) even after nasopharyngeal swab returned negative and absence of gastrointestinal symptoms. A recent large meta-analysis of 60 studies (4243 patients) demonstrated a 48.1% [95% CI (confidence interval), 38.3%-57.9%] pooled prevalence of SARS-CoV-2 RNA detection in the stool (38.5% with diarrhea and 8.7% without diarrhea) [12]. In this study, 70.3% of patients were positive for viral RNA in stool collected even after resolution of respiratory symptoms and a noted negative nasopharyngeal swab for the virus, indicating prolonged viral shedding in the gastrointestinal tract [12].

Taken together, the presence of the ACE2 receptor as well as TMPRSS2 or cathepsins on the surface of enterocytes, the presence of viral RNA localized within enterocytes, the active release of viral particles from enterocytes, and its subsequent detection within the stool suggest a direct cytopathological effect of SARS-CoV-1 and thus of SARS-CoV-2. Indeed, post-mortem analyses of SARS-CoV-1 patients demonstrated the presence of mild inflammatory changes with associated architectural disruption of brush-border enterocytes [8]. This architectural disruption is hypothesized to lead to impaired enterocyte function, local malabsorption, and a loss of intestinal permeability, thus leading to diarrhea [3, 11]. In addition, the incidence of diarrhea in cases of SARS-CoV-1 and SARS-CoV-2 could be secondary to altered kinetics of the ACE2 receptor, which has been previously shown to uptake dietary amino-acids and promote gut homeostasis, with ACE2 alterations associated with colitis [11]. Furthermore, the presence of an extensive lymphoid network within the gut, and its critical role in intestinal hemostasis coupled with pathological data demonstrated widespread degenerative changes in intestinal lymphoid tissues and lymphoid depletion suggests an additional or alternative etiology of intestinal malabsorption and the prevalence of diarrhea amongst SARS-CoV-2 infected patients. 

In this case series, two patients demonstrated diarrhea as the predominant symptom, with a lack of associated respiratory symptomatology. The gastrointestinal symptoms in these patients occurred 3.5-7.5 days after the onset of fever seemingly affording an appropriate temporal relationship from the onset of infection, respiratory replication, and viral dissemination to infect enterocytes and lymphocytes of gut and shedding of virus in the stool [10]. Among gastrointestinal symptoms, the commonly reported symptoms are nausea/vomiting 7.8% (95% CI, 7.1%-8.5%), diarrhea 7.7% (95% CI, 7.2%-8.2%), and mild abdominal pain 2.7% (95% CI, 2.0%-3.4%) [5]. In another study, anorexia (34.8%), diarrhea (33.7%), and nausea/vomiting (26.4%) were reported as common gastrointestinal symptoms in the US population [17]. A significantly high rate of fatigue (65%), myalgia (49.2%), sore throat (21.5%), anosmia, and ageusia (16.9%) was associated in patients with predominant gastrointestinal symptomatology [17]. While the presence of viral shedding in the stool has been previously noted and prior studies have suggested the possibility of fecal-oral transmission, data remain scarce [8, 10]. It is highly probable that the gastrointestinal manifestations are a secondary phenomenon stemming from secondary viral sepsis and carriage to the intestinal endothelial and epithelial system through the blood after primary infection of the respiratory epithelium, from circulating lymphocytes and altered gut-associated lymphoid tissues, or the elaboration of a local or systemic cytokine cascade leading to intestinal permeability. Nevertheless, the detection of viral particles via RT-PCR of stool samples, even after resolution of respiratory symptomatology and intriguingly, gastrointestinal symptoms, coupled with the high false-negative rate and user-dependent collection techniques in nasopharyngeal sampling, stool testing may be a more sensitive and specific means of SARS-CoV-2 infected patients as well as asymptomatic carriers, particularly in patients with high clinical suspicion with a negative COVID-19 test. 

Of note, hepatocyte involvement and liver injury have also been reported in recent clinical studies on COVID-19 with a rate noted to be as high as 50% of cases, a feature also shared with the original SARS-CoV-1 pandemic [2, 18]. A meta-analysis on 47 studies (10,890 patients) demonstrated elevated liver enzymes with the pooled prevalence of AST 15% (95% CI 13.6%-16.5%) and ALT 15% (95% CI 13.6%-16.4%) [5]. In these studies, the most common findings were mild to moderately elevated ALT and AST as well as occasional direct hyperbilirubinemia [18]. In our case series, one patient was found to have moderately elevated ALT, AST, and alkaline phosphate with associated normal bilirubin (Case 3). Of note, in SARS-CoV-1 infection, liver biopsy demonstrated a significant increase in mitotic cells with associated hepatocyte ballooning with intracellular eosinophilic bodies suggestive of hepatocellular injury, degeneration, and apoptosis; whereas post-mortem analysis noted degenerative changes in the centrilobular zone [19]. Additional pathologic manifestations, from SARS-COV-1 demonstrated mild inflammation in the portal tract, sinusoidal lymphocytosis, and mild cellular hydropic degeneration (mainly in the perivenular areas) in the hepatic parenchyma [20]. Intriguingly, SARS-CoV-1 RNA was detected within hepatocytes. However, hepatocytes and the endothelial lining of the sinusoids were shown to lack ACE2 expression, though surface staining of the bile canaliculi and cholangiocytes was observed [16]. In this paradigm, however, one would expect an elevated alkaline phosphatase and direct hyperbilirubinemia, a feature rarely seen in SARS-CoV1 and SARS-CoV2 infection, which when coupled with the presence of viral RNA sequences in hepatocytes and pathology samples demonstrating hepatocyte degeneration suggesting hepatocyte infection via an alternative receptor, or hepatocyte injury through a secondary process, such as downstream immunological responses to the virus. This contrasts with other coronaviruses, such as MERV-CoV, whose functional receptor, DDP-4, is highly expressed on hepatocytes. At this time the exact mechanism of liver injury in patients with COVID-19 remains unknown, however, SARS-CoV-2 seems to cause liver injury by cell-mediated apoptosis versus hepatocyte steatosis and portal and lobular infiltration. Post-mortem biopsies from a deceased COVID-19 patient showed moderate microvesicular steatosis and mild portal and lobular activity, findings that mirror those observed with SARS-CoV and MERS-CoV liver injury. 

## Conclusions

Typically, patients with COVID-19 present with a respiratory syndrome. Nevertheless, recent data suggest that gastrointestinal symptoms (anorexia, diarrhea, abdominal pain) could be the predominant, if not the sole manifestation of SARS-CoV-2 infection, like prior studies with SARS-CoV-1. As such, unexplained diarrheal illness or elevated liver enzymes, even in the absence of respiratory symptomatology, should prompt consideration for SARS-CoV-2 infection and a diagnosis of COVID-19. Furthermore, given the presence of SARS-CoV-2 viral shedding in the stool in multiple patient subpopulations - SARS-CoV-2 infected patients with predominantly respiratory symptoms, SARS-CoV-2 infected patients with predominantly gastrointestinal symptoms, and in SARS-CoV-2 infected patients that have recovered from their illness, coupled with the technical issues with nasopharyngeal swabbing as well as the high false-negative rate of the test, stool testing for SARS-CoV-2 should be considered as a mainstay of diagnosis. There is a possibility of delayed viral transmission through the fecal-oral route, suggesting that healthcare personnel should take extra precautions while collecting stool samples or performing endoscopic procedures. While therapeutic strategies targeting ACE2 and TMPRSS2 could prevent viral cell binding, the finding of viral RNA within hepatocytes that notably lack the ACE2 receptor, coupled with the presence of viral RNA in cells lacking TMPRSS2, suggest an alternative receptor-binding site as well as an alternative cell-surface protease facilitating viral entry. Thus, strategies targeting the viral S-protein or the downstream inflammatory cytokine cascade may be paramount to prevent and limit SARS-CoV-2 infection. Further clinical trials are needed to understand the route of viral transmission, pathophysiology of gastrointestinal manifestations, and treatment strategies.
